# Long-Term Prognosis and Antimycobacterial Glycolipid Antibody as Biomarker in Mycobacterium avium-intracellulare Complex Pulmonary Disease

**DOI:** 10.1128/spectrum.00530-22

**Published:** 2022-04-25

**Authors:** Ryoji Maekura, Keisuke Miki, Yoshitaka Tateishi, Sohkichi Matsumoto, Seigo Kitada, Mari Miki, Hiroshi Kida

**Affiliations:** a Department of Respiratory Medicine, National Hospital Organization Osaka Toneyama Medical Center, Toyonaka, Japan; b Graduate School of Medical Safety Management, Jikei University of Health Care Sciences, Osaka, Japan; c Department of Bacteriology, Niigata University Graduate School of Medicine, Niigata, Japan; d Kitada Respiratory Clinic, Yao, Japan; e Tokushima Prefecture Naruto Hospital, Tokushima, Japan; Johns Hopkins University School of Medicine

**Keywords:** mycobacterial glycolipid (trehalose-6, 6-dimycolate), glycopeptidolipid core, prognostic survival predictor, *Mycobacterium avium-intracellulare* complex pulmonary disease

## Abstract

Clinical characteristics and outcomes of multidrug chemotherapy have been used as the main prognostic factors for Mycobacterium avium-intracellulare complex pulmonary disease (MAC-PD) over the last decade; however, no useful prognostic biomarkers have been reported. The aim is to ascertain whether the serum antibody titers could include useful prognostic predictors of MAC-PD. Ninety-four patients with MAC-PD were enrolled and regularly followed up with for more than 5 years or until death. Cox proportional hazard regression and receiver operating characteristic (ROC) curve analyses were used to identify predictors of mortality in this prospective observational study. According to treatment outcomes, 85 patients completed follow-up and were classified into four groups. Seventeen patients (20%) died during follow-up (median, 10.1 years; interquartile range, 8.1 to 12.4 years). All 11 patients with MAC-PD-specific death were included in the 14 patients of the group nonresponsive to the multidrug chemotherapy. They had significantly higher anti-*Mycobacterium* glycolipid (MBGL) antibody titers than those in the other groups and a significantly (*P* < 0.0001) poorer survival prognosis. The anti-MBGL antibody titers also served as a negative prognostic factor. A cutoff score of 7, which was calculated by clinical poor prognostic characteristics and anti-MBGL antibody titers, differentiated the nonresponse group and the other groups at baseline (sensitivity, specificity, and area under the curve: 92.9%, 81.7%, and 0.95, respectively). In conclusion, anti-MBGL antibody titers were useful to assess the refractory MAC-PD. The predictions of treatment outcome and mortality become more accurate by using anti-MBGL antibody and clinical poor prognostic characteristics together.

**IMPORTANCE** The natural history of MAC-PD is challenging to predict in immunocompetent patients at diagnosis, and the current multidrug chemotherapy options are not strong enough to eliminate mycobacteria from the lungs. Therefore, the diagnosis of MAC-PD does not necessarily lead to the decision to start chemotherapy. We have also observed refractory patients in clinical practice, who were resistant to multiple-drug chemotherapy and showed persistent excretion of MAC bacilli and progressive worsening of chest radiographic findings until death. We have reported that the measurements of anti-MBGL antibody titers helped assess refractory MAC-PD in this study. Furthermore, the predictions of treatment outcome and mortality become more accurate by using the anti-MBGL antibody in addition to clinical poor prognostic characteristics, which were older age, lower body mass index, the positive results of a smear test for acid-fast bacteria (AFB), and presence of cavitary disease.

## INTRODUCTION

Mycobacterium avium-intracellulare complex (MAC) comprises bacteria that are ubiquitous in nature and have been isolated from water, soil, residential bathrooms, and other environmental sources (1–5). The prevalence of MAC pulmonary disease (MAC-PD) is increasing in Japan and worldwide (6–8). The natural history of MAC-PD is difficult to predict in immunocompetent patients at diagnosis, and the current multidrug chemotherapy options are not strong enough to eliminate mycobacteria from the lungs (9, 10). Therefore, the diagnosis of MAC-PD does not necessarily lead to the decision to start chemotherapy. In clinical practice, we have also observed that nonresponsive patients, who were resistant to multiple-drug chemotherapy, showed persistent excretion of MAC bacilli and progressive worsening of chest radiographic findings until death (11). The cured patients who responded to chemotherapy and exhibited a negative sputum culture conversion had a good prognosis. Some patients maintained a stable clinical and radiographic picture for 10 years without treatment (12, 13).

Clinical characteristics at diagnosis that were relevant prognostic factors for poorer outcomes in patients with MAC-PD, such as male sex, older age, lower body mass index (BMI), presence of cavitary disease, and higher erythrocyte sedimentation rate (ESR), have been reported over the past decade (14–18). Large and irregular cavities with consolidation strongly correlated with disease progression, leading to respiratory failure and high mortality rates (11, 19).

We had previously developed two commercial enzyme-linked immunosorbent assay (ELISA) kits that detect specific mycobacterial infection by measuring the titers of IgG-antibody against *Mycobacterium* glycolipid (MBGL), including cord factor (trehalose-6,6′-dimycolatethe), and minor glycolipids as well as specific MAC infection by measuring the titer of IgA antibody against the glycopeptidolipid core (GPLcore) which is specifically present in the cell walls of MAC and rapidly growing nontuberculous mycobacteria (20–23). The anti-GPLcore antibody levels decreased in response to multidrug chemotherapy; however, the changes were not a good predictor of therapeutic outcomes as a biomarker (24). The cavitary lesions were associated with anti-MBGL antibody titers in pulmonary tuberculosis but not with anti-GPLcore antibody titers in MAC-PD as reported previously (21, 23). Therefore, we conducted this prospective, observational study to ascertain whether the follow-up measurements of both antibody titers were useful to discriminate between refractory patients who were nonresponsive to multidrug chemotherapy and other patients with MAC-PD.

## RESULTS

### Characteristics and treatment outcomes.

**FIG 1:**
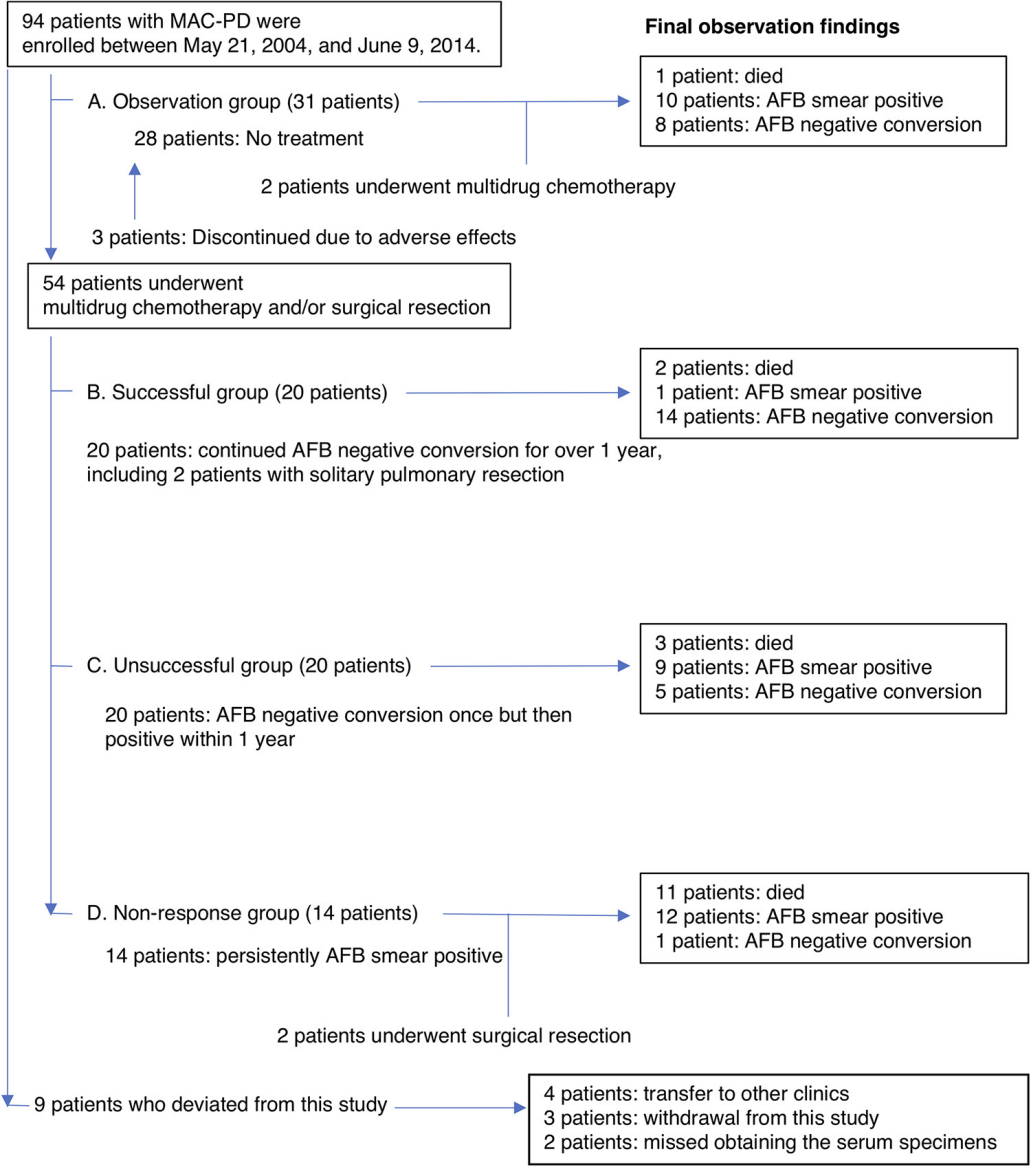
Design of the prospective observational study in a clinical practice covered by Japanese health care insurance. AFB, acid-fast bacteria.

A total of 85 of the 94 enrolled patients with MAC-PD were followed up with for more than 5 years or until death and were classified into four groups according to the treatment outcome: group A, observational group, 31 patients; group B, successful group, 20 patients; group C, unsuccessful group, 20 patients; and group D, nonresponse group, 14 patients. The remaining nine patients deviated from the study design and were not included in the analysis. Clinical characteristics, mycobacterial species, changes in bacteriological and chest radiographic findings, and survival prognosis during follow-up (median, 10.1 years; interquartile range, 8.1 to 12.4 years) are shown in Table 1 and Fig. 1. In total, 54 (63.5%) patients underwent treatment according to the guidelines, and their initial, secondary, and additional multidrug chemotherapy regimens are shown in Table S1 (25, 26). The initial sputum culture conversion rate was high (74%), but the recurrence rate within 1 year was also high (50%). Sputum culture-negative conversions continued after treatment to the final visits of 14 (70%) patients of the successful group. However, sputum culture-positive reconversions were observed in 6 (30%) patients after treatment during a 10-year follow-up. Five (25%) patients of the unsuccessful group and 8 (26%) patients of the observational group observed culture-negative conversions at the final visits. However, the sputum smear and culture positives for acid-fast bacteria (AFB) were observed in 10 (32%) and 23 (74%) patients of the observational group after long-term observation (10.6 [8.9 to 12.6] years). Two patients underwent the initial multidrug chemotherapy 6 and 9 years after enrollment.

**TABLE 1:** Characteristics, chest radiographic findings, and survival prognosis of patients with Mycobacterium avium-intracellulare pulmonary disease according to outcome of treatment

Characteristic	Value(s) for treatment outcome group:				Total
	Observational group	Successful group	Unsuccessful group	Nonresponse group	
No. of patients (%)^*a*^	*n* = 31 (36.5)	*n* = 20 (23.5)	*n* = 20 (23.5)	*n* = 14 (16.5)	*n* = 85
Male:female	6:25	2:18	4:16	3:11	15:70
Age (yrs)^*b*^	69.0 (62.6–77.3)	68.0 (65.5–71.8)	61.2 (57.0–69.4)	70.2 (63.7–80.3)	68.4 (61.4–73.2)
BMI^*b*^	19.9 (18.7–21.6)	17.5 (16.3–21.1)	18.6 (17.4–21.6)	16.3 (15.3–18.5)	18.7 (16.5–20.8)
ESR (mm/h)^*b*^	24 (19–41.5)	41 (25.5–77.3)	20.5 (16.5–37.8)	64.5 (49.8–71.3)	31 (20–59)
Follow-up duration (yrs)^*b*^	10.6 (8.9–12.6)	11.7 (8.8–15.1)	9.7 (8.4–10.7)	7.4 (6.1–14.5)	10.1 (8.1–12.4)
No. of hospitalizations^*b*^	0 (0–1)	1 (1–1)	2 (1–3)	4.5 (3.8–6.3)	1 (0–3)
Underlying lung diseases					
Previous tuberculosis^*a*^	2	4	1	3	10 (11.8)
COPD^*a*^	3	0	2	2	7 (8.2)
Interstitial pneumonia^*a*^	0	1	0	2	3 (3.5)
Comorbidity					
Diabetes mellitus^*a*^	2 (6.5)	2 (10)	1 (5)	7 (50)	12 (14.1)
Cardiovascular disease^*a*^	2	3	4	4	13 (15.3)
Hyperlipidemia^*a*^	3	5	3	1	12 (14.1)
GERD^*a*^^,^^*c*^	2	2	5	4	13 (15.3)
Mycobacterial species					
M. avium^*a*^	21	14	14	11	60 (70.6)
M. intracellulare^*a*^	8	4	5	3	20 (23.5)
Both^*a*^	2	2	1	0	5 (5.9)
Concurrent M. abscessus^*a*^	2	0	3	0	5 (5.9)
Concurrent *Aspergillus*^*a*^	3 (9.7)	2 (10)	3 (15)	5 (35.7)	13 (15.3)
Sputum smear test positivity for AFB					
(+/−) at enrollment^*a*^	8 (26)/23	3 (15)/17	9 (45)/11	11 (78.6)/3	31 (36.5)/54
(+/−) at final visit^*a*^	10 (32)/21	1 (5)/19	9 (45)/11	12 (85.7)/2	34 (40)/51
Sputum culture-negative conversion^*a*^	8 (25.8)	14 (70)	5 (25)	1 (7.1)	28 (32.9)
Chest radiographic lesions					
Type (NC-NB/C-NB/FC)					
At enrollment	28/3/0	19/1/0	16/2/2	5/2/7	68/8/9
At final visit	26/4/1	17/3/0	15/4/1	0/6/8^*f*^	58/17/10
Extent (minimal/moderately/far advanced)					
At enrollment	28/3/0	11/9/0	7/13/0	1/11/2	47/36/2
At final visit	25/6/0	8/12/0	6/13/1	0/3/11	39/34/12
Progression of radiographic findings^*d*^					
Stable/slightly progressive/progressive	20/8/3	9/7/4	10/1/9	0/0/14	39/16/30
Causes of death^*e*^					
MAC-specific^*a*^	0	0	0	11	11 (12.9)
MAC-nonspecific causes^*a*^	1	2	3	0	6 (7.1)

a Numbers are presented as value (percentage).

b Numbers are presented as median (interquartile range).

c BMI, body mass index; ESR, erythrocyte sedimentation rate; COPD, chronic obstructive pulmonary disease; GERD, gastroesophageal reflux disease; AFB, acid-fast bacteria; MAC-PD, Mycobacterium avium-intracellulare complex pulmonary disease; NC-NB, noncavitary nodular bronchiectatic disease; C-NB, cavitary nodular bronchiectatic disease; FC, fibrocavitary type.

d Progression of radiographic findings was graded on a three-level scale: stable, shrinkage or unchanged of abnormal shadows; slightly progressive, a slight increase in the size of preexisting abnormal shadows; progressive, emergence of consolidation or cavitary lesions in addition to an increase in the size preexisting abnormal shadows.

e MAC-nonspecific causes: hemoptysis with Aspergillosis in observational group, heart disease with pneumonia and lung cancer in successful group, COPD with MAC-PD (2 patients), and aortic aneurysm rupture in unsuccessful group.

f Including 2 patients with giant cavity.

Seventeen patients (20%) died from all causes of death in this study. All 11 patients who died because of MAC-PD were included in the nonresponse group. In this group, the patients were characterized by low BMI, high ESR, a high comorbidity rate of diabetes mellitus, and frequent detection of *Aspergillus*. Despite repeated chemotherapy, the results of smear tests for AFB were always strongly positive, and the extent of lesions in the radiographic findings gradually progressed and spread beyond the unilateral lung field, with cavity lesions being detectable in all patients (Table 1). In the nonresponse group, the isolates had acquired clarithromycin resistance in 6 patients at enrollment and in 8 other patients during follow-up.

### Comparisons of serum antibody titers among groups divided by treatment outcomes, sputum bacterial status, and survival prognosis.

The titer changes of anti-GPLcore and anti-MBGL antibodies for 10 years from enrollment for the observational, successful, unsuccessful, and nonresponse groups, according to the measurement range of the kits, are shown in Fig. S1.

**FIG 2:**
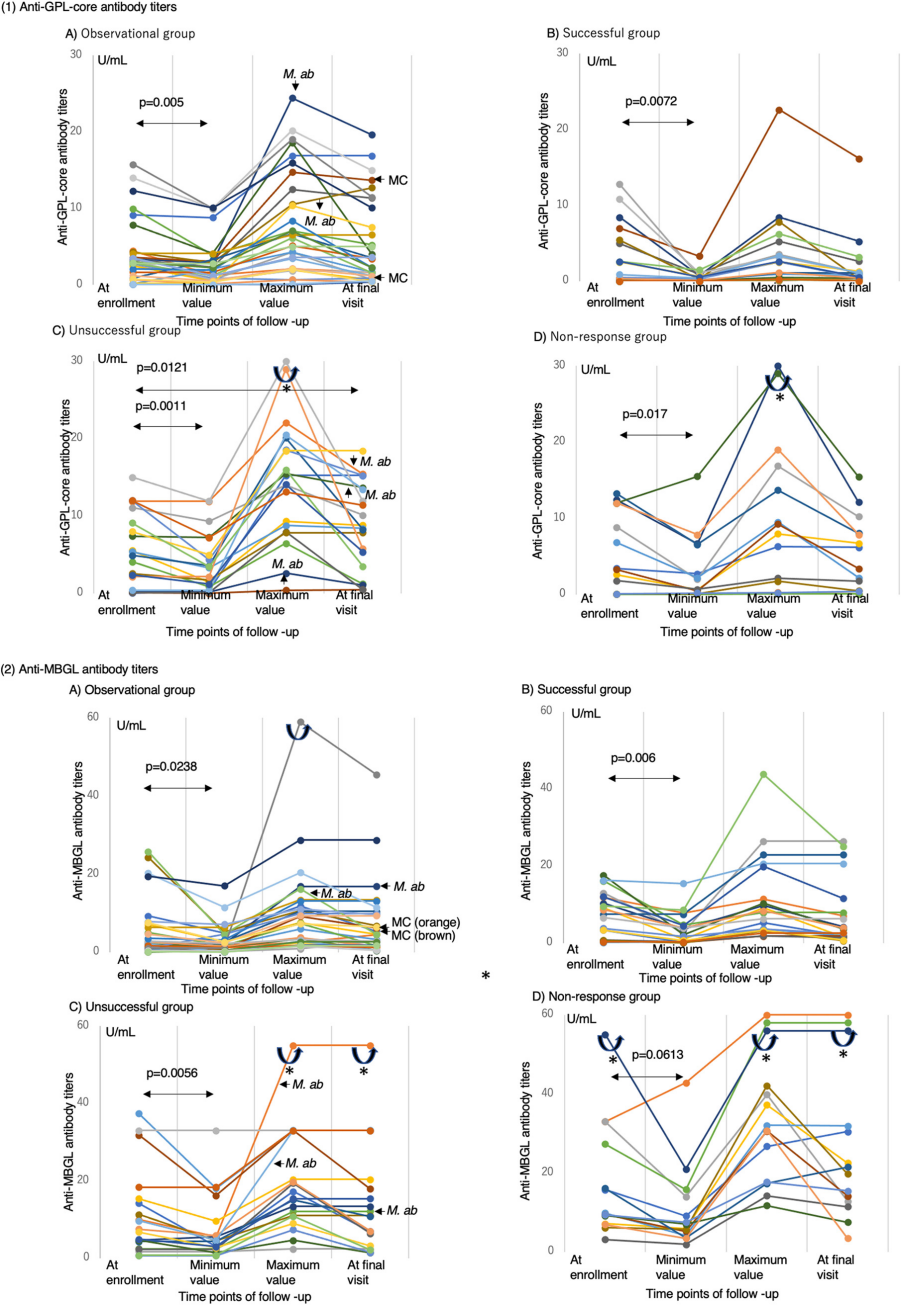
Comparisons among (1) anti-MAC antibody titers and (2) anti-MBGL antibody titers at four time points of follow-up based on treatment outcomes. (1) Anti-GPLcore antibody titers: (A) anti-GPL-core antibody titers of individual patients in the observational group (*n* = 31), (B) anti-GPL-core antibody titers of individual patients in the successful group (*n* = 20), (C) anti-GPL-core antibody titers of individual patients in the unsuccessful group (*n* = 20), and (D) anti-GPL-core antibody titers of individual patients in the nonresponse group (*n* = 14). *M. ab* indicates patients with concurrent Mycobacterium abscessus infection; MC indicates patients who underwent multidrug chemotherapy. Curved arrows with “*”: the upper points over this mark indicate more than 30 U/mL of anti-GPLcore antibody titer. (2) Anti-MBGL antibody titers: (A) anti-MBGL antibody titers of individual patients in the observational group (*n* = 31), (B) anti-MBGL antibody titers of individual patients in the successful group (*n* = 20), (C) anti-MBGL antibody titers of individual patients in the unsuccessful group (*n* = 20), (D) anti-MBGL antibody titers of individual patients in the nonresponse group (*n* = 14). *M. ab* indicates patients with concurrent M. abscessus infection; MC indicates patients who underwent multidrug chemotherapy. Curved arrows with “*”: the upper points over this mark indicate more than 60 U/mL of anti-MBGL antibody titer.

The titers of anti-GPLcore and anti-MBGL antibodies were statistically analyzed at four time points (at enrollment, minimum measured value, maximum measured value, and at final visit) during follow-up. The results of the comparison among the groups are shown in Table 2 [Table tab3]to 4. Anti-MBGL antibody titers, unlike anti-GPLcore antibody titers, were significantly higher in the nonresponse group than in the other groups. The titers of anti-MBGL antibody in patients with persistent sputum culture positivity despite treatment were also significantly higher than those in patients with sputum-negative conversion at the final visit, regardless of the presence or absence of treatment. Additionally, anti-MBGL antibody titers in MAC-PD-specific nonsurvivors were significantly higher than those in survivors. The minimum anti-GPLcore antibody titers were significantly lower than the anti-GPLcore antibody titer measured at enrollment in all groups (Fig. 2). The minimum anti-MBGL antibody titers were also significantly lower than the anti-MBGL antibody titer measured at enrollment in all groups except the nonresponse group.

**TABLE 2:** Comparisons of serum antibody titers using anti-GPLcore antibody and anti-MBGL antibody among patients by treatment outcome group^*a*^

Characteristic	Value(s)^*b*^ for treatment outcome group:				K–W test *P* value	Dunn test *P* value(s)	Total
	Observational group	Successful group	Unsuccessful group	Nonresponse group			
No. of patients	*n* = 31	*n* = 20	*n* = 20	*n* = 14			*n* = 85
Anti-GPLcore antibody (U/mL)							
At the time of enrollment	3.0 (1.2–4.3)	0.7 (0.06–5.3)	5.2 (0.8–10.6)	3.3 (0.07–12.0)	0.1544		2.9 (0.4–7.6)
Minimum measured value	1.9 (0.7–9.7)^+^	0.3 (0.02–0.7)*	2.7 (0.5–6.7)*^,+^	1.3 (0.2–6.6)	0.0006	*, 0.0012; ^+^, 0.0017	1.2 (0.3–3.3)
Maximum measured value	6.1 (2.1–12.5)^+^	2.5 (0.4–4.8)*^,+^	14.7 (8.2–19.7)*	8.6 (1.4–17.4)	0.0001	*, <0.0001; ^+^, 0.0431	6.6 (2.1–15.0)
Final measured value	3.4 (1.2–10.1)^+^	0.6 (0.4–1.3)*	8.6 (4.0–13.6)*^,+^	4.8 (0.5–8.6)	0.0002	*, <0.0001; ^+^, 0.0150	3.2 (0.7–9.5)
Anti-MBGL antibody (U/mL)							
At the time of enrollment	2.1 (0.5–7.1)*	8.1 (1.5–12.0)	7.2 (3.0–15.1)	9.7 (7.1–28.7)*	0.0008	*, 0.0007	6.6 (1.7–12.0)
Minimum measured value	1.7 (0.5–4.6)*	2.4 (0.4–4.7)^+^	4.4 (2.4–8.7)	6.5 (4.0–14.5)*^,+^	0.0003	*, 0.0007; ^+^, 0.0080	3.0 (1.4–5.9)
Maximum measured value	7.5 (2.7–11.8)*^,#^	8.2 (3.4–17.8)^+^	16.4 (10.9–33.0)^#^	31.4 (17.6–51.4)*^,+^	<0.0001	*, <0.0001; ^+^, 0.0003; ^#^, 0.0066	11.1 (4.2–21.8)
Final measured value	4.6 (1.7–9.9)*	3.8 (1.8–10.7)^+^	11.6 (4.0–29.9)	20.6 (12.5–39.4)*^,+^	0.0001	*, 0.0004; ^+^, 0.0016	7.0 (2.5–17.4)

a The symbols “*,” “+,” and “#” are indicated to compare the difference in the Dunn test. K–W test, Kruskal–Wallis test.

b Values are presented as median (interquartile range).

**TABLE 3 tab3:** Comparisons of serum antibody titers using anti-GPLcore antibody and anti-MBGL antibody among patients by sputum bacterial status^*a*^

Characteristic	Value(s)^*b*^ for patients with sputum bacterial status:				K–W test *P* value	Dunn test *P* value(s)
	Negative conversion with no treatment	Negative conversion with treatment	Occasional positive with treatment	Persistent positive with treatment		
No. of patients	*n* = 8	*n* = 20	*n* = 35	*n* = 22		
Anti-GPLcore antibody (U/mL)						
At the time of enrollment	1.6 (0.3–3.0)	2.5 (0.1–7.7)	3.0 (0.9–7.0)	3.5 (1.5–9.6)	0.481	
Minimum measured value	0.5 (0.1–0.7)	0.4 (0.1–0.9)*	2.1 (0.9–4.1)*	2.1 (0.5–6.6)	0.0045	*, 0.0118
Maximum measured value	1.9 (1.0–6.3)	2.9 (0.6–7.9)	7.9 (3.3–16.0)	10.0 (3.3–18.9)	0.0142	
Final measured value	0.7 (0.4–4.4)	0.6 (0.4–2.3)*^,#^	5.0 (1.2–11.5)^#^	6.5 (1.7–12.9)*	0.0003	*, 0.0046; ^#^, 0.0026
Anti-MBGL antibody (U/mL)						
At the time of enrollment	2.8 (0.3–6.6)*	5.5 (2.4–11.5)	2.7 (0.7–11.3)^#^	9.8 (7.4–24.7)*^,#^	0.0005	*, 0.0044; ^#^, 0.0014
Minimum measured value	1.7 (0.3–2.6)*	2.4 (0.7–4.0)^#^	2.7 (0.8–6.4)^+^	5.8 (4.5–10.3)*^,#,+^	0.0002	*, 0.0013; ^#^, 0.0026; ^+^, 0.0115
Maximum measured value	3.6 (2.0–7.5)*	10.0 (4.1–18.3)^#^	10.5 (2.8–20.4)^+^	28.7 (13.6–40.4)*^,#,+^	<0.0001	*, <0.0001; ^#^, 0.0096; ^+^, 0.0019
Final measured value	2.3 (1.6–4.3)*	3.8 (1.8–9.9)^#^	9.4 (2.6–16.9)	17.6 (7.4–33.0)*^,#^	0.0001	*, 0.0010; ^#^, 0.0013

a The symbols “*,” “+,” and “#” are indicated to compare the difference in the Dunn test.

b Values are presented as median (interquartile range).

**TABLE 4:** Comparisons of serum antibody titers using anti-GPLcore antibody and anti-MBGL antibody among patients by survival prognosis^*a*^

Characteristic	Value(s)^*b*^ for:			K–W test *P* value	Dunn test *P* value
	Survivors	Nonsurvivors, MAC-nonspecific	Nonsurvivors, MAC-specific		
No. of patients	*n* = 68	*n* = 6	*n* = 11		
Anti-GPLcore antibody (U/mL)					
At the time of enrollment	2.8 (0.5–6.6)	1.4 (0.1–10.5)	3.4 (0.1–12.0)	0.6371	
Minimum measured value	1.2 (0.4–3.3)	0.5 (0.1–5.9)	2.0 (0.2–6.6)	0.8371	
Maximum measured value	6.5 (2.1–14.6)	4.8 (2.1–14.6)	9.3 (1.7–16.7)	0.9144	
Final measured value	2.9 (0.7–8.7)	3.2 (0.4–15.3)	3.4 (0.5–10.2)	0.9749	
Anti-MBGL antibody (U/mL)					
At the time of enrollment	4.5 (1.1–9.7)*	11.0 (4.9–14.7)	9.8 (9.2–33)*	0.0046	*, 0.0094
Minimum measured value	2.6 (0.8–5.2)*	4.6 (2.7–5.5)	7.0 (4.1–14.0)*	0.006	*, 0.0059
Maximum measured value	9.8 (3.3–19.6)*	15.4 (7.8–28.7)	30.6 (17.3–42.1)*	0.001	*, 0.0008
Final measured value	5.0 (1.9–13.3)*	9.8 (5.6–23.8)	19.7 (12.8–31.9)*	0.0018	*, 0.0015

a The symbol “*” is indicated to compare the difference in the Dunn test.

b Values are presented as median (interquartile range).

The results using receiver operating characteristic (ROC) curve analyses of both antibody titers are shown in Table S2.

Sensitivity and specificity were 92.9% and 58.8% with the cutoff point 6.1 U/mL of anti-MBGL antibody titer at enrollment, and sensitivity and specificity were 100% and 74.5% with the cutoff point 11.7 U/mL of MBGL antibody titer at maximum value for differentiating the nonresponse group from the observational and successful groups (Table S2A). Sensitivity and specificity were 81.8% and 70.3% with the cutoff point 9.2 U/mL of anti-MBGL antibody titer at enrollment, and sensitivity and specificity were 100% and 61.8% with the cutoff point 11.7 U/mL of anti-MBGL antibody titer at maximum value for differentiating MAC-PD-specific nonsurvivors from survivors (Table S2C).

### Comparisons of serum antibody titers among groups classified by chest radiographic findings.

Table 5 [Table tab6]to 7 show a comparison of serum antibody titers measured at four time points among patients with different types, extents, and progressions of chest radiographic lesions. Anti-MBGL antibody titers in patients with fibrocavitary (FC) lesions at enrollment were significantly (*P* = 0.046) higher than those in patients with noncavitary nodular bronchiectatic type (NC-NB) lesions, unlike the anti-GPL core antibody. In patients who had developed the FC lesion at the final visit, the anti-MBGL antibody titers at all four time points were significantly higher than those in patients with NC-NB lesions, especially at enrollment (*P* = 0.0047). In patients whose extent of lesions was far advanced at the final visit, the anti-MBGL antibody titers at all four time points were significantly higher than those in patients with a minimal extent of lesions. The anti-MBGL antibody titers, unlike the anti-GPL-core antibody titers, in patients classified as the progressive group at all four time points were significantly higher than those in patients in the stable group.

**TABLE 5:** Comparisons of anti-GPLcore and anti-MBGL antibody titers of different types of chest radiographic lesions

Characteristic	Value(s)^*a*^ for patients with lesion type:			K–W test *P* value	Dunn test *P* value
	NC-NB	C-NB	FC		
Information from patients at enrollment					
No. of patients	*n* = 68	*n* = 8	*n* = 9		
Anti-GPLcore antibody (U/mL)	2.6 (0.3–5.4)	4.3 (0.7–7.3)	8.8 (2.5–12.2)	0.1699	
Anti-MBGL antibody (U/mL)	5.0 (1.1–10.2)*	8.2 (3.7–14.0)	15.7 (6.2–32.4)*	0.0339	*, 0.046
Information from patients who had a final visit					
No. of patients	*n* = 58	*n* = 16	*n* = 11		
Anti-GPLcore antibody (U/mL)					
At the time of enrollment	2.8 (0.6–5.9)	2.9 (0.3–8.8)	3.4 (0.1–12.0)	0.8689	
Minimum measured value	1.3 (0.4–3.3)	0.6 (0.2–4.6)	1.2 (0.1–6.6)	0.956	
Maximum measured value	6.6 (2.4–14.0)	7.2 (1.2–16.7)	6.3 (1.7–16.9)	0.9837	
Final measured value	2.9 (0.9–9.1)	1.9 (0.5–9.5)	3.6 (0.5–10.2)	0.8984	
Anti-MBGL antibody (U/mL)					
At the time of enrollment	4.5 (1.0–10.6)*	7.1 (3.1–9.8)	15.7 (7.8–33.0)*	0.0053	*, 0.0047
Minimum measured value	2.5 (0.7–4.8)*	3.1 (2.2–5.7)	7.2 (5.2–16.2)*	0.0017	*, 0.0013
Maximum measured value	9.3 (3.1–17.8)*	12.0 (8.0–31.7)	30.6 (14.2–42.1)*	0.0006	*, 0.0008
Final measured value	6.4 (1.8–13.2)*	5.0 (3.0–19.5)	17.9 (11.4–0.4)*	0.0045	*, 0.0038

a NC-NB, noncavitary nodular bronchiectatic type; C-NB, cavitary nodular bronchiectatic type; FC, fibrocavitary type. The symbol “*” is indicated to compare the difference in the Dunn test.

**TABLE 6 tab6:** Comparisons of anti-GPLcore and anti-MBGL antibody titers of different extents of chest radiographic lesions

Characteristic	Value(s)^*a*^ for patients with lesion extent:			K–W test *P* value	Dunn test *P* value
	Minimal	Moderate	Far advanced		
Information from patients at enrollment					
No. of patients at enrollment	*n* = 47	*n* = 36	*n* = 2		
Anti-GPLcore antibody (U/mL)	3.0 (0.9–5.4)	2.5 (0.1–9.8)	8.8, 12.4	0.1545	
Anti-MBGL antibody (U/mL)	2.7 (0.7–7.6)*^,#^	9.4 (5.4–15.6)^#^	33.0, 75.9*	<0.0001	*, 0.014; ^#^, 0.0003
Information from patients who had a final visit					
No. of patients at final visit	*n* = 39	*n* = 34	*n* = 12		
Anti-GPLcore antibody (U/mL)					
At the time of enrollment	3.0 (0.9–5.4)	2.4 (0.1–8.3)	7.8 (2.2–12.0)	0.1161	
Minimum measured value	1.4 (0.5–3.1)	0.6 (0.1–3.4)	2.5 (0.5–6.6)	0.0859	
Maximum measured value	6.6 (2.6–12.5)	4.9 (1.1–14.4)	11.6 (3.2–18.5)	0.2799	
Final measured value	3.4 (1.1–11.4)	1.2 (0.5–8.8)	4.9 (1.9–9.7)	0.3065	
Anti-MBGL antibody (U/mL)					
At the time of enrollment	2.1 (0.5–7.8)*^,#^	7.6 (4.8–13.3)^#^	9.7 (7.5–28.8)*	0.0002	*, 0.0023; ^#^, 0.0024
Minimum measured value	1.6 (0.4–4.6)*^,#^	4.0 (2.3–5.9)^#^	6.1 (3.5–12.8)*	0.0017	*, 0.0058; ^#^, 0.0213
Maximum measured value	7.4 (2.7–13.1)*^,#^	15.3 (7.6–33)^#^	28.7 (15.0–37.9)*	<0.0001	*, 0.0001; ^#^, 0.0039
Final measured value	4.2 (1.5–11.0)*^,#^	7.5 (3.5–23.5)^#^	14.8 (8.5–31.5)*	0.0011	*, 0.0034; ^#^, 0.0199

a The symbols “*,” and “#” are indicated for each pair to compare the difference in the Dunn test.

**TABLE 7:** Comparisons of anti-GPLcore and anti-MBGL antibody titers of different progressions of chest radiographic lesions

Characteristic	Value(s)^*a*^ for patients with lesion progression:			K–W test *P* value	Dunn test *P* value
	Stable	Slightly progressive	Progressive		
No. of patients	*n* = 39	*n* = 15	*n* = 31		
Anti-GPLcore antibody (U/mL)					
At the time of enrollment	2.8 (0.2–5.6)	3.6 (2.5–9.1)	3.1 (0.4–8.8)	0.4516	
Minimum measured value	1.2 (0.1–3.6)	1.1 (0.5–3.0)	1.2 (0.4–3.5)	0.9833	
Maximum measured value	5.1 (1.1–14.0)	8.4 (3.2–14.7)	8.0 (1.9–16.0)	0.5341	
Final measured value	1.7 (0.5–8.3)	3.2 (0.7–13.7)	3.6 (0.8–8.4)	0.6616	
Anti-MBGL antibody (U/mL)					
At the time of enrollment	2.7 (0.6–8.7)*	9.3 (1.5–13.0)	9.2 (6.1–17.6)*	0.0013	*, 0.001
Minimum measured value	1.7 (0.5–4.6)*	3.2 (0.8–4.7)	5.2 (2.7–9.1)*	0.0014	*, 0.0011
Maximum measured value	7.5 (2.6–16.2)*	9.7 (6.3–17.3)	19.7 (11–33.0)*	0.0003	*, 0.0002
Final measured value	3.3 (1.6–11.5)*	6.3 (4.2–11.6)	14.1 (6.5–30.4)*	0.0008	*, 0.0006

a Progression of radiographic findings were graded on a three-level scale: stable, shrinkage of or no change in abnormal shadows; slightly progressive, a slight increase in the size of preexisting abnormal shadows; progressive, emergence of consolidation or cavitary lesions in addition to an increase in the size preexisting abnormal shadows. The symbol “*” is compare the difference in the Dunn test.

### Survival prognosis predictors.

**FIG 3:**
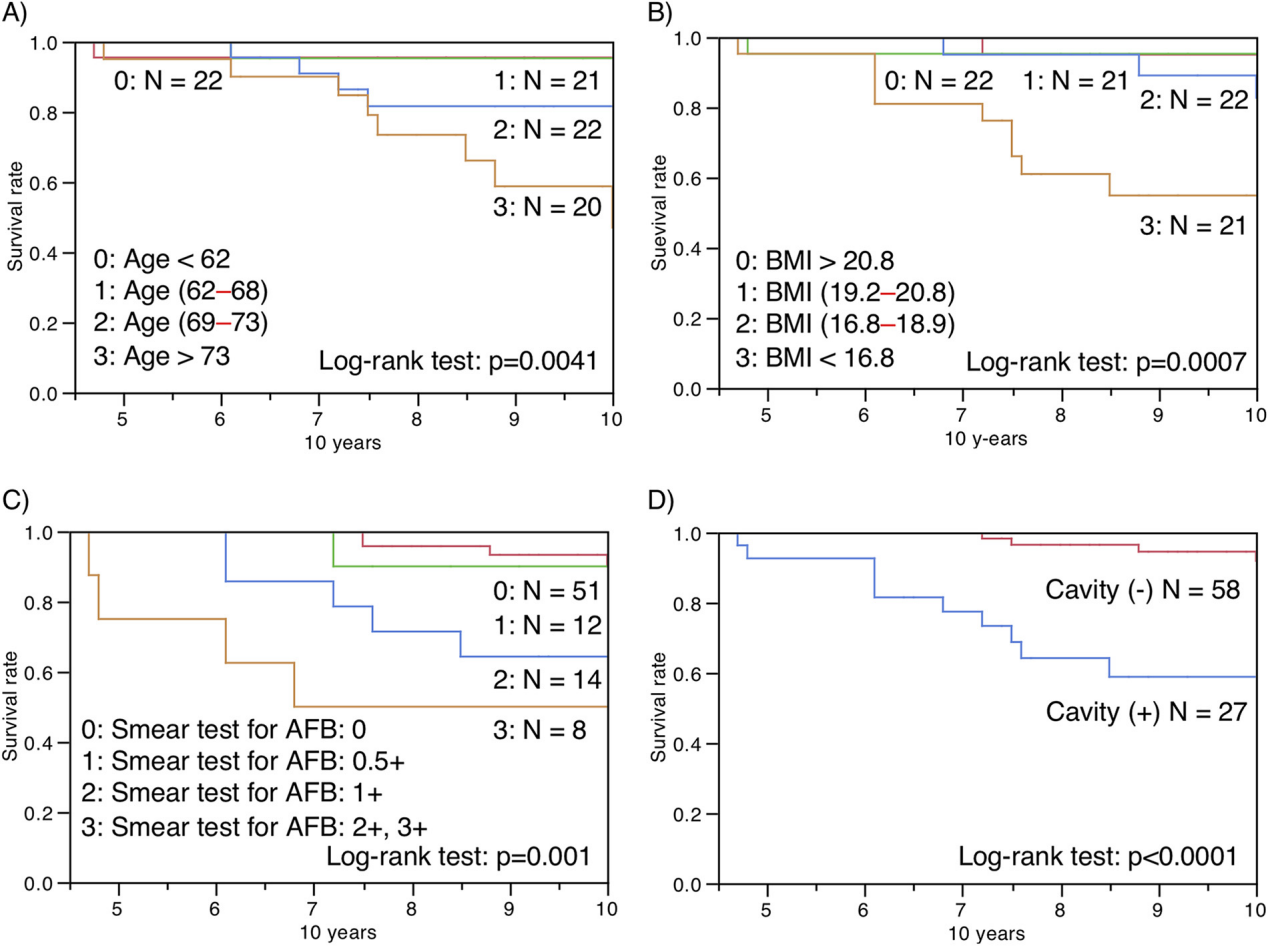
Kaplan–Meier survival analyses of patients with MAC-PD divided by (A) age, (B) body mass index (BMI), (C) sputum smear test results for acid-fast bacteria (AFB) at enrollment, and (D) presence of cavity during follow-up, for all-cause mortality.

**FIG 4:**
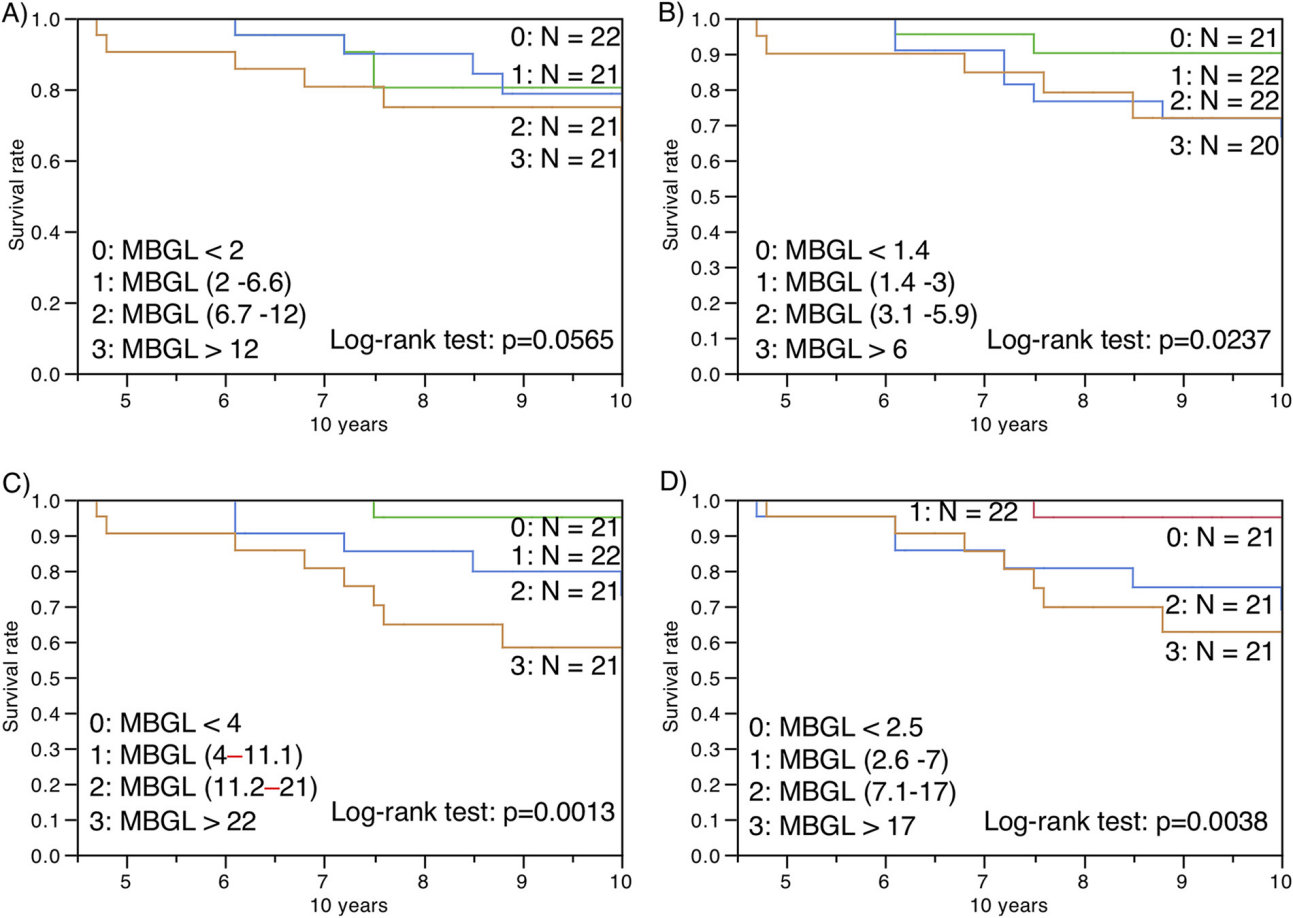
Kaplan–Meier survival analyses of patients with MAC-PD divided by (A) anti-MBGL antibody titers at enrollment, (B) minimum anti-MBGL antibody titers, (C) maximum anti-MBGL antibody titers, and (D) anti-MBGL antibody titers at the final visit for all-cause mortality.

**FIG 5:**
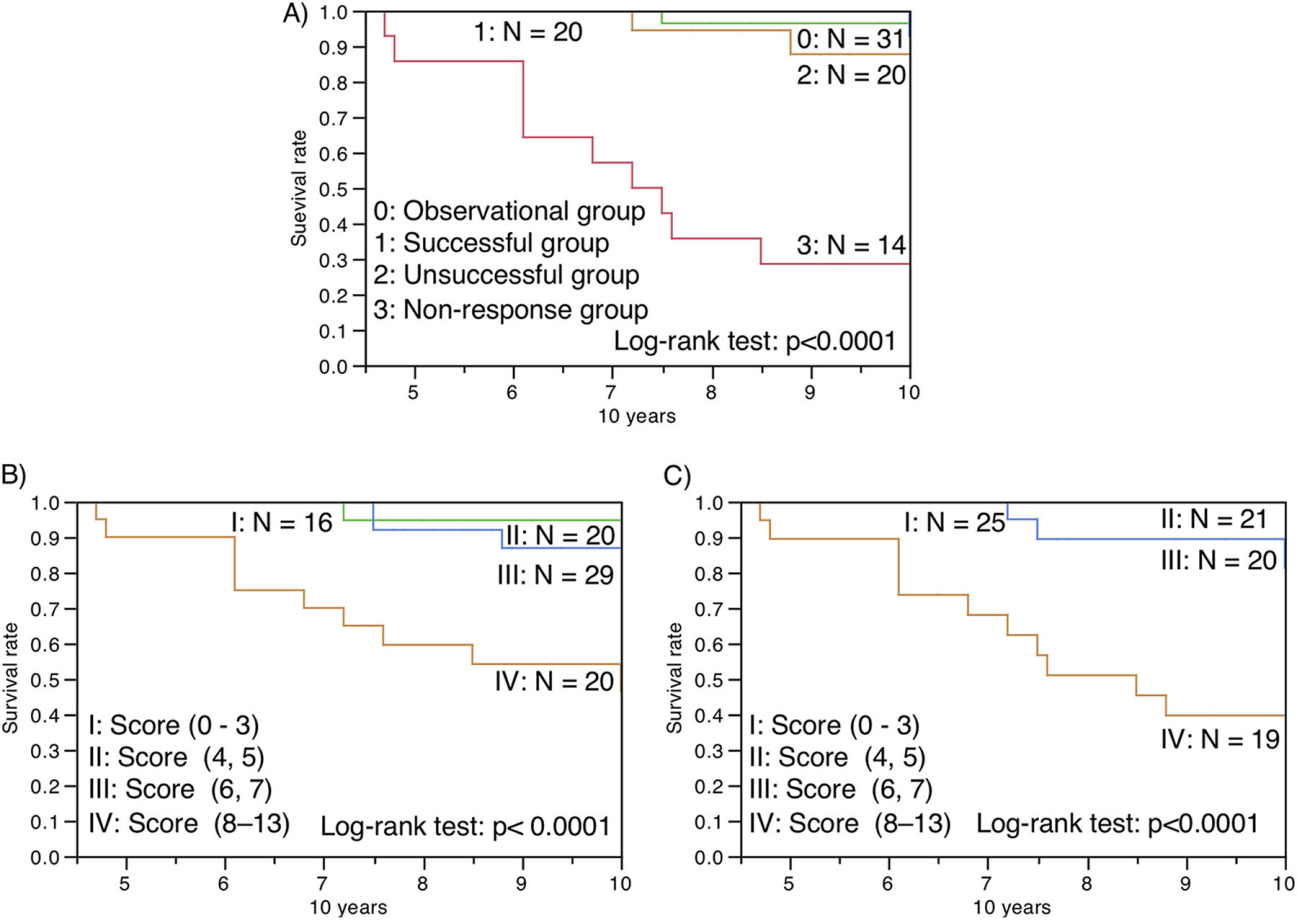
Kaplan–Meier survival analyses of patients with MAC-PD divided by (A) treatment outcomes and the scores using (B) anti-MBGL antibody titers at enrollment and (C) maximum anti-MBGL titers, in addition to negative prognostic factors (age, BMI, cavity, and sputum smear test for AFB), for all-cause mortality.

The univariate and multivariate Cox proportional hazard models showed that the clinical characteristics, which were older age, lower BMI, higher ESR, comorbidities of diabetes or gastroesophageal reflux disease, smear positivity for AFB, concurrent aspergillosis, far advanced extent, and cavitation on chest radiography, were negative prognostic factors for all-cause mortality in MAC-PD patients (Table 8, Fig. 3). The anti-MBGL antibody titers, unlike the anti-GPL-core antibody titers, were also negative prognostic factors independent of age, BMI, ESR, and cavitary lesions (Table 9). Patients with anti-MBGL antibody titers above 11 or 22 during follow-up have an abysmal prognosis (Fig. 4). The patients in the nonresponse group had a significantly (*P* < 0.0001) poorer survival prognosis than those in the other groups (Table 10, Fig. 5A). However, only two patients died within the first 5 years.

**TABLE 8:** Univariate analysis of the risk of all-cause mortality

Variable	Risk ratio	95% CI^*a*^	*P* value
Age (yrs)	1.145	1.059–1.255	0.0005
Sex	1.385	0.311–4.53	0.6311
BMI	0.654	0.5–0.831	0.0002
ESR (mm/h)	1.022	1.005–1.038	0.0121
Comorbidity			
Diabetes mellitus	6.761	2.153–20.598	0.0017
GERD	3.932	1.182–11.857	0.0274
Bacteriological findings			
Smear test at enrollment	3.599	1.824–7.102	0.0004
Smear test at final visit	4.884	2.5–9.591	<0.0001
Sputum bacterial status	8.899	3.187–36.921	<0.0001
Concurrent M. abscessus	0.305	0.082–1.973	0.1789
Concurrent *Aspergillus*	5.432	1.745–16.387	0.0047
Radiographic lesions			
Cavity at enrollment	8.376	2.786–27.789	0.0002
Cavity at final visit	10.011	3.037–44.892	<0.0001
Extent at enrollment			
Moderate, minimal	6.888	1.815–44.81	0.0032
Advanced, minimal	125.9	13.38–1238	0.0003
Advanced, moderate	18.28	2.481–96.84	0.0082
Extent at final visit			
Moderate, minimal	4.695	0.695–91.78	0.1183
Advanced, minimal	51.39	9.55–950.8	<0.0001
Advanced, moderate	10.95	3.517–40.79	<0.0001
Progression of lesions	2.711	1.376–6.329	0.0031
Anti-GPLcore antibody (U/mL)			
Value at enrollment	1.098	0.981–1.221	0.0999
Minimum measured value	1.106	0.964–1.24	0.1374
Maximum measured value	1.038	0.986–1.082	0.1411
Final measured value	1.033	0.935–1.128	0.5029
Anti-MBGL antibody (U/mL)			
Value at enrollment	1.045	1.013–1.072	0.0097
Minimum measured value	1.057	1.003–1.1	0.0394
Maximum measured value	1.031	1.012–1.047	0.0022
Final measured value	1.033	1.011–1.053	0.0046

a CI, confidence interval; GERD, gastroesophageal reflux disease.

**TABLE 9:** Multivariate analysis of the risk of all-cause mortality

Variable	Risk ratio	95% CI	*P* value
Group 1			
Age	1.084	0.994–1.199	0.0709
BMI	0.688	0.476–0.920	0.0076
ESR	1.01	0.984–1.034	0.4493
Cavity	3.698	1.091–12.856	0.036
Variables at enrollment	1.038	1.006–1.067	0.0238
Group 2			
Age	1.071	0.982–1.182	0.1283
BMI	0.688	0.466-0.924	0.0081
ESR	1.01	0.983–1.036	0.4634
Cavity	5.844	1.829–20.566	0.0031
Anti-MBGL antibody maximum value	1.025	1.007–1.042	0.0095

**TABLE 10:** Analysis between nonresponse group and other three groups by treatment outcome

Group/reference group	Risk ratio	95% CI	*P* value
Nonresponse/observational	36.2	6.87–666.7	<0.0001
Nonresponse/successful	24.9	4.71–459.7	<0.0001
Nonresponse/unsuccessful	11.1	2.91–72.8	0.0002

**FIG 6:**
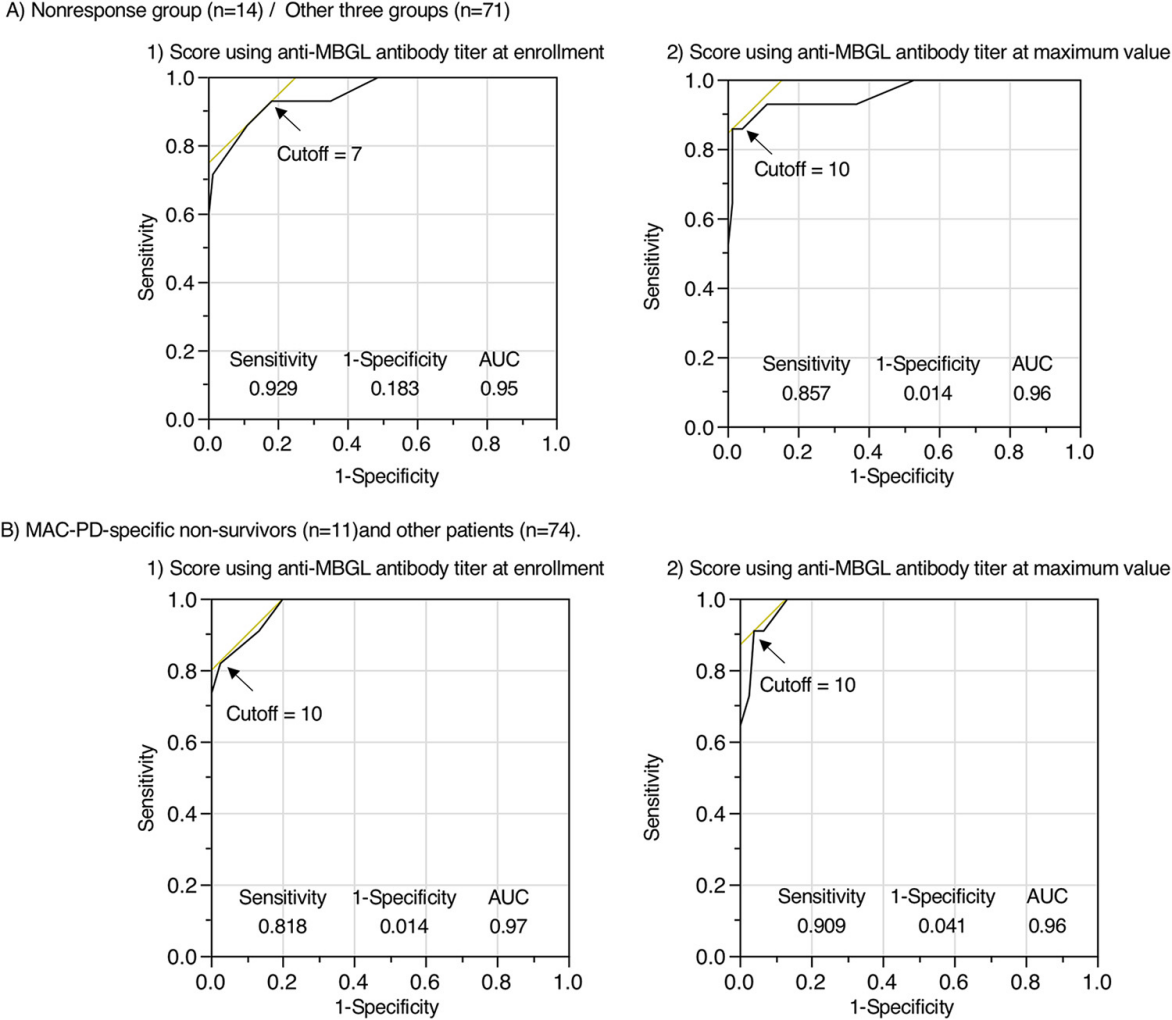
ROC (receiver operating characteristic) curve analyses of the score for differentiating between (A) the nonresponse group and the other three groups and (B) the MAC-PD-specific nonsurvivors and the other patients. Age, BMI, smear test results, and anti-MBGL antibody titers were divided into quartiles and scored. The cavity was divided into presence and absence and scored. The scores are shown in Table S3. The cutoff point was determined as the antibody titer showing the highest [sensitivity − (1 − specificity)] value. Age, BMI, smear test results, and anti-MBGL antibody titers were divided into quartiles and scored. The cavity was divided into presence and absence and scored. The scores are shown in Table S3. The cutoff point was determined as the antibody titer showing the highest [sensitivity − (1 − specificity)] value. AUC, area under the curve.

Furthermore, the patients were divided into four groups based on quartiles of age, BMI, the results of a smear test for AFB, anti-MBGL antibody titer, and presence or absence of a cavitary lesion and scored (Table S3). Both highest-scored groups (IV: score 8 to 13) showed a significantly (*P* < 0.0001) worse survival prognosis for all-cause mortality using anti-MBGL titer at enrollment and maximum measurement value (Table 11, Fig. 5B and C). The results using ROC curve analyses of the score are shown in Fig. 6. Sensitivity and specificity were 92.9% and 81.7% with the cutoff point score 7 at enrollment, and sensitivity and specificity were 85.7% and 98.6% with the cutoff point score 11 at the maximum value of anti-MBGL antibody titer for differentiating the nonresponse group (*n* = 14) from the other three groups (*n* = 71). Sensitivity and specificity were 81.8% and 98.6% with the cutoff point score 7 at enrollment, and sensitivity and specificity were 90.9% and 95.9% with the cutoff point score 11 at the maximum value of anti-MBGL antibody titer for differentiating MAC-PD-specific nonsurvivors (*n* = 11) from other patients (*n* = 74). The univariate and multivariate Cox proportional hazard model analyses for MAC-PD-specific mortality are shown in Table S4.

**TABLE 11:** Univariate analyses of score

Variable^*a*^	Risk ratio	95% CI	*P* value
Using anti-MBGL antibody titers at enrollment			
Score	1.855	1.487–2.396	<0.0001
Score group	4.386	2.221–11.284	<0.0001
Using maximum anti-MBGL antibody titers			
Score	1.993	1.565–2.682	<0.0001
Score group	7.422	3.047–28.244	<0.0001

a Scores were calculated by age, BMI, smear test results, cavity, and anti-MBGL antibody titers and are shown in Table S3.

## DISCUSSION

In this study, 85 (90%) of the 94 enrolled patients regularly visited the respiratory clinic of the National Toneyama Hospital for a long time. Fifty-two patients underwent the appropriate initial multidrug chemotherapy for more than 1 year, according to the guideline (25, 26). The culture conversion rate of 74% in response to the initial treatment was consistent with the results from previous reports (27–29). Patients included in both the unsuccessful and nonresponse groups underwent aggressive additional multidrug chemotherapies, including aminoglycosides and fluoroquinolones, based on the results of susceptibility testing to antimycobacterial agents in patients’ isolates (30). At the final visit, the culture-negative conversion rates were very low (25%) in the unsuccessful group. However, the number of deaths in the first 5 years after enrollment was 2 (5-year mortality: 2.4%); this mortality rate is much lower than the previously reported rates (12.4% to 35%) (11, 17, 18, 28, 31–34).

The 14 patients in the nonresponsive group had a significantly worse survival prognosis than the other groups. MAC-PD caused all 11 deaths in this group, including those of the 7 patients with underlying lung diseases or chronic pulmonary aspergillosis (35). MAC-PD may be associated with respiratory failure in chronic obstructive pulmonary disease (COPD) and with hemoptysis in chronic pulmonary aspergillosis in other groups. Therefore, it is difficult to define MAC-PD-specific death accurately. However, it is essential to accurately differentiate refractory patients from other MAC-PD patients with good survival prognoses within a short period from diagnosis. Numerous studies have reported that older age, lower BMI, higher ESR, underlying lung disease, diabetes comorbidity, sputum smear positive for AFB, concurrent detection of *Aspergillus*, extensive spread of radiographic lesions, and the presence of cavitary lesions were negative prognostic factors for mortality from all causes (11, 14–18, 32–35). The results of this study were consistent with previous reports. The increase of anti-MBGL antibody titer also was an adverse prognostic factor. The score calculated by this antibody, in addition to the above negative prognostic factors for mortality, could accurately differentiate the nonresponders to the multidrug chemotherapy from other patients with good survival prognosis at an early stage of follow-up. The anti-MBGL antibody titers, specifically for detecting mycobacterial infection, have been found to be a negative prognostic factor independent of age, BMI, ESR, and cavity lesions, unlike the anti-GPL-core antibody titers. These results can be explained by the fact that the anti-MBGL antibody titer was significantly high in patients refractory to treatment, had persistent AFB-positive results during follow-up, and was thus determined to differentiate MAC-PD-specific nonsurvivors. Furthermore, this antibody titer was also significantly higher in patients whose radiographic lesions progressively worsened and spread to a far higher extent in those patients than in patients with stable radiographic lesions. In particular, the patients with FC lesions at the final visit had already shown significantly higher antibody titers at enrollment than patients with NC-NB or cavitary nodular bronchiectatic-type (C-NB) lesions. The elevated anti-MBGL antibody titer reflected the exacerbation of MAC-PD. We have previously reported the associations between anti-MBGL antibody titer and radiographic lesions in pulmonary tuberculosis (21).

The anti-MBGL antibody titer was measured as the serum level of IgG against cord factor (trehalose-6,6-dimycolate) as the main component. Yamamura et al. previously reported that experimental lung cavity formation could be induced in rabbit lungs by injection of mycobacterial protein mixed with cord factor but not the individual components (36, 37). The detection of the anti-MBGL antibody was considered to reflect the presence of cord factor in the lung lesion. The anti-GPL-core antibody titer was not a good prognostic predictor because it showed no significant difference between patients with NC-NB lesions and those with FC lesions, although it may reflect disease activity, as in previous studies (24).

The seropositive rates of anti-GPL-core and anti-MBGL antibodies at enrollment were 72.9% and 74.1%, respectively, similar to those reported in previous studies. They increased to 85.9% and 92.9%, respectively, during follow-up (21, 23, 38). The exacerbation of lung lesions, indicated by the expansion of lesions and appearance of cavities, and the sputum smear test persistently positive for AFB might be associated with the increase in seropositive rates during follow-up, as in patients with chronic and active pulmonary TB (20). A seronegative result for both antibodies was observed in only one patient who exhibited IgG positivity for lipoarabinomannan. The antibodies for other mycobacterial antigens (such as antigen 85 complex proteins, heparin-binding hemagglutinin, and mycobacterial DNA-binding protein 1) with biological activity were also detectable in the sera of patients with MAC-PD (no data shown). Both anti-GPL-core and anti-MBGL antibodies can be used to assess disease activity and as prognostic and predictive biomarkers rather than serodiagnosis tools for MAC-PD. In addition, the measurement of other antibodies expressed during mycobacterial growth or dormancy as biomarkers may be able to assess the stage spectrum of MAC infection (advanced disease to active disease to subclinical infection to quiescent infection).

The geographical and host-dependent variability in the genetic diversity of Mycobacterium avium has been demonstrated using minimum spanning tree and multidimensional scaling analyses based on the variable number of tandem repeats data (39). The genetic results of the isolates from humans in Japan showed less similarity to the isolates from France and Finland. Extensive mutual homologous recombination also occurred among the five major lineages of M. avium subsp. *hominissuis* in the search for local adaptation mechanisms (40). At the start of this study, the detection rate of serotypes in isolates from MAC-PD patients decreased from 62% to 36% (particularly 59% to 3% for serovar 4) compared to that in our previous study (11). Therefore, M. avium bacilli might alter their characteristics depending on the environment.

This study was conducted in a clinical practice using Japanese health care insurance at a single facility. The overall study period was very long (10.1 [8.1 to 12.4] years) since it was time-consuming to enroll a number of cases in the nonresponse group sufficient to reach the target sample size and complete the study. The low 5-year mortality rate may be due to a shortage of patients in the nonresponse group. The lower mortality rate may be attributed to the following effects. (i) Aggressive additional chemotherapies (Table S1). Amikacin inhalation and gatifloxacin were effective in patients with MAC-PD, but the Japanese health care insurance did not cover amikacin, and the sale of gatifloxacin was discontinued. Amikacin liposome inhalation for refractory MAC-PD has recently been covered by insurance and may improve the effects of treatment (41). (ii) Prevention of reinfection from residential bathrooms. Most patients in this study were married, full-time housewives. The recovery rate of MAC isolates from their residential bathrooms was significantly higher (52%) than that (2%) from bathrooms of healthy volunteers (4, 5). Based on these results, we had instructed them to wear a mask while cleaning the bathroom to prevent reinfection. Prevention of reinfection may also have contributed to improving the survival prognosis. (iii) Decrease in the proportion of the cavity form. The proportions of the NB form also significantly increased from 60% to 84.4% among MAC-PD patients between circa 2000 and 2016 in another Japanese institute (42). (iv) Decrease in the number of patients with previous tuberculosis. The rates of patients with previous tuberculosis (11.8%) and cavity lesions (20%) in this study were lower than those (62% and 62%, respectively) reported previously (5-year mortality: 28%) (11). (v) Surgical intervention. The culture-negative conversion was observed in one of two patients with surgical resection of the lung in the nonresponse group. (vi) Pulmonary rehabilitation, including occupational therapy (43). The advanced MAC-PD patients with breathlessness underwent pulmonary rehabilitation. However, further research is needed to clarify these issues.

### Conclusion.

Anti-MBGL antibody level, unlike anti-GPLcore antibody, reflected the severity of the pathological condition and was a good predictor of mortality as a biomarker. Therefore, the predictions of treatment outcome and mortality become more accurate by using anti-MBGL antibody and clinical poor prognostic characteristics together.

## MATERIALS AND METHODS

### Study design and patients.

This prospective observational study was conducted in a clinical practice covered by Japanese health care insurance. We enrolled 94 patients with MAC-PD between May 2004 and June 2014 who regularly visited the clinic of the National Toneyama Hospital and were followed up with until 30 September 2020. The diagnosis of MAC-PD in immunocompetent patients was based on the criteria advocated by the ATS 1997 (25) and ATS/IDSA 2007 (26). Patients underwent the initial and additional (as needed) multidrug chemotherapies according to recent guidelines available at the time of management (25, 26). The initial multidrug chemotherapy (three- or four-drug regimens with the new macrolide antibiotics) commenced at admission after the potential side effects, low chemotherapy success rate, and risk of recurrence of multidrug chemotherapy were discussed with individual patients, and the regimen was confirmed after ascertaining that no problematic side effects would occur. Additional multidrug chemotherapy regimens were determined according to the guidelines and based on susceptibility assay results for antimycobacterial agents in patients’ isolates. The chemotherapy continued for at least 12 months in the respiratory clinic of the National Toneyama Hospital. Patients visited the clinic monthly during chemotherapy and were followed up with at 3-, 6-, or 12-month intervals after treatment and until death.

This study was approved by the National Toneyama Hospital Review Board, Osaka, Japan (approved on 21 May 2004: no. 0315). All patients provided written informed consent. The trial protocol was registered at UMIN Clinical Trials Registry (https://upload.umin.ac.jp/cgi-open-bin/ctr_e/ctr_his_list.cgi?recptno=R000051999; ID: UMIN000045542).

The 94 enrolled patients were classified into four groups according to the treatment outcome during follow-up: (i) the observational group comprised patients who did not receive treatment, (ii) the successful group comprised patients who underwent multidrug chemotherapy or surgical resection and whose sputum cultures for acid-fast bacteria (AFB) were consecutively negative for more than 12 months, (iii) the unsuccessful group comprised patients demonstrating sputum culture-negative conversion during multidrug chemotherapy treatment but showed sputum culture-positive conversion within 12 months, and (iv) the nonresponse group comprised patients who did not respond to multidrug chemotherapy and remained persistently smear positive for AFB. The following were collected at enrollment and each visit during follow-up: sputum bacteriological results for AFB, chest radiography (including high-resolution computed tomography [HRCT] as needed), and serum antibody levels (anti-MBGL and anti-GPLcore antibodies). For the treatment, the target sample sizes of (i) successful and unsuccessful groups and (ii) the nonresponse group were determined to be 28 and 13 patients, respectively, for 10-year survival analysis, based on previous findings (two-tailed α = 0.05, β = 0.17, q_1_ = 0.31, relative hazard = 0.375) (11). Despite treatment, some patients worsened from MAC-PD and progressed to respiratory failure requiring long-term oxygen therapy. The cause of death was a MAC-PD-specific death when patients consistently showed positive smear culture results.

### Examinations.

The concentrated sputum smear tests were performed using fluorochrome stain auramine-O dyes and Ziehl-Neelsen stain. The cultures used a *Mycobacterium* growth indicator tube (Japan Becton, Dickinson Company, Tokyo, Japan) or the conventional method with 2% Ogawa egg medium (Kyokuto Pharmaceuticals, Tokyo, Japan). Mycobacterial species, such as M. avium and M. intracellulare, were identified with a DNA-DNA colorimetric microdilution plate hybridization kit (DDH Mycobacteria; Kyokuto Pharmaceuticals, Tokyo, Japan) as described previously (11). Anti-MBGL (previously named TBGL) IgG and anti-GPLcore IgA titers were measured using commercial ELISA kits (Tauns Laboratory, Inc., Shizuoka, Japan) according to the manufacturer’s instructions (21, 23). The cutoff points and upper measurement limits were 2 and 32 U/mL for the anti-MBGL antibody and 0.7 and 14 U/mL for the anti-GPLcore antibody, respectively. The values exceeding the upper measurement limit of the kits were measured by increasing the dilution ratio of the serum with phosphate-buffered saline containing 1% bovine serum albumin.

The type of chest radiographic lesion was determined to be noncavitary nodular bronchiectatic (NC-NB), cavitary nodular bronchiectatic (C-NB), or fibrocavitary (FC), based on the HRCT results. The extent was classified into three groups (minimal, moderate, or far advanced) according to the criteria of pulmonary tuberculosis (44). Progression of radiographic findings was graded on a three-level scale described in previous articles (13): stable, defined as shrinkage or unchanged abnormal shadows; slightly progressive, defined as a slight increase in the size of preexisting abnormal shadows; progressive, defined as the emergence of consolidation or cavitary lesions in addition to an increase in the size of preexisting abnormal shadows. The radiographic findings were evaluated independently by two pulmonologists and a radiologist, and the final diagnosis was decided by consensus.

### Statistical analysis.

Statistical analyses were performed using conventional computer analysis software (JMP 9, SAS Institute Inc, Cary, NC, USA). Reported values are consistently expressed as numbers and percentages or medians and interquartile ranges. The Kruskal-Wallis test was used to determine differences in physiologic parameters between the groups. Differences between pairs of groups were analyzed with the Dunn test. Cox proportional hazard regression and Kaplan–Meier analyses were used to identifying predictors of mortality. Statistical significance was evaluated using the log-rank test. We also performed receiver operating characteristic (ROC) curve analyses for predicting the worst survival prognosis and treatment outcome. Differences were considered significant at *P* < 0.05.
