# Supplementary figures and images for: Long-Term Prognosis and Antimycobacterial Glycolipid Antibody as Biomarker in Mycobacterium avium-intracellulare Complex Pulmonary Disease
Source: Microbiol Spectr. 2022 Apr 25;10(3):e00530-22. doi: 10.1128/spectrum.00530-22 (PMC9241601; doi:10.1128/spectrum.00530-22)

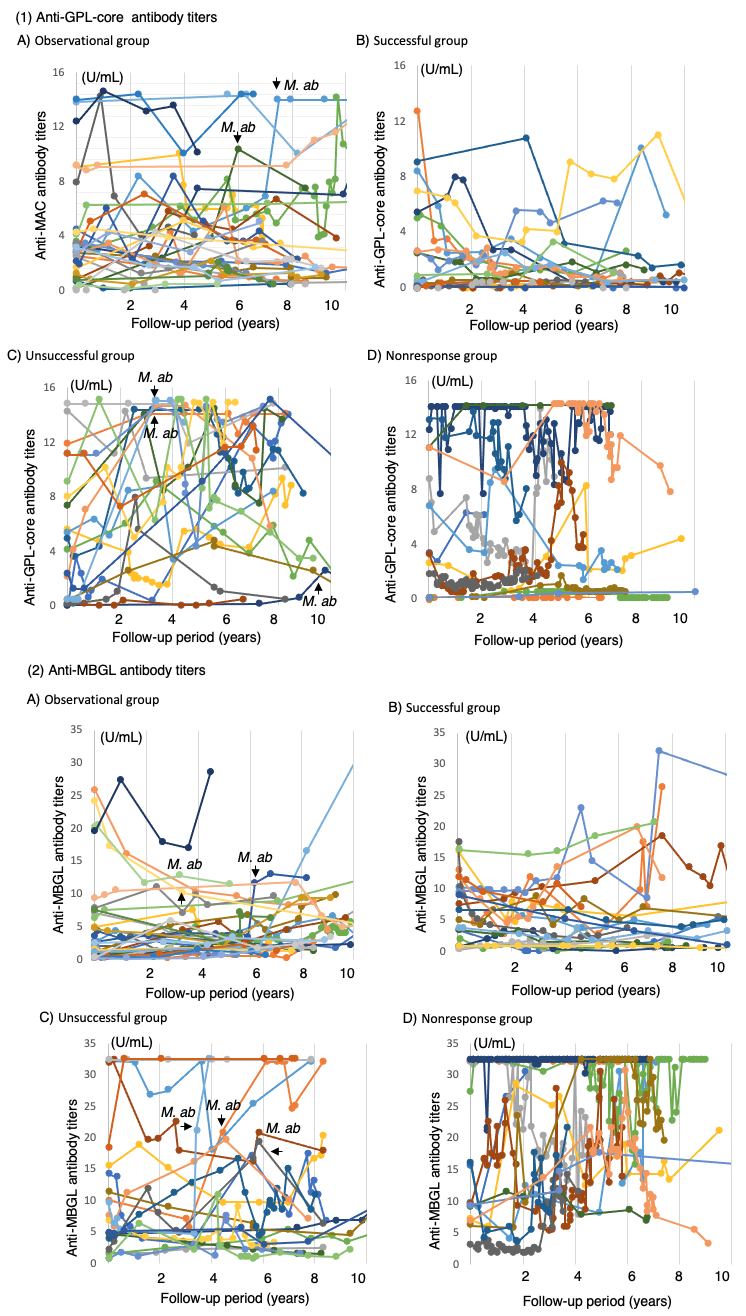

Supplement: SUPPLEMENTAL FILE 2 — Supplemental material. Download spectrum.00530-22-s002.tif, TIF file, 3.7 MB [file spectrum.00530-22-s002.tif]
